# Toenail mineral concentration and risk of esophageal squamous cell carcinoma, results from the Golestan Cohort Study

**DOI:** 10.1002/cam4.1247

**Published:** 2017-11-10

**Authors:** Maryam Hashemian, Gwen Murphy, Arash Etemadi, Hossein Poustchi, John D. Brockman, Farin Kamangar, Akram Pourshams, Masoud Khoshnia, Abdolsamad Gharavi, Sanford M. Dawsey, Paul. J. Brennan, Paolo. Boffetta, Azita Hekmatdoost, Reza Malekzadeh, Christian C. Abnet

**Affiliations:** ^1^ Digestive Oncology Research Center Digestive Diseases Research Institute Tehran University of Medical Sciences Tehran Iran; ^2^ Metabolic Epidemiology Branch Division of Cancer Epidemiology and Genetics National Cancer Institute Bethesda Maryland; ^3^ Departments of Nutrition and Biochemistry Faculty of Medicine Sabzevar University of Medical Sciences Sabzevar Iran; ^4^ Liver and Pancreatobiliary Diseases Research Center Digestive Diseases Research Institute Tehran University of Medical Sciences Tehran Iran; ^5^ Research Reactor Centre University of Missouri‐Columbia Columbia Missouri; ^6^ Digestive Disease Research Center Digestive Diseases Research Institute Tehran University of Medical Sciences Tehran Iran; ^7^ School of Computer, Mathematical, and Natural Sciences Morgan State University Morgan State University Baltimore Maryland; ^8^ Golestan Research Center of Gastroenterology and Hepatology Golestan University of Medical Sciences Gorgan Iran; ^9^ International Agency for Research on Cancer Lyon France; ^10^ Tisch Cancer Institute Icahn School of Medicine at Mount Sinai New York New York; ^11^ Departments of Clinical Nutrition and Dietetics Faculty of Nutrition and Food Technology National Nutrition and Food Technology Research Institute Shahid Beheshti University of Medical Sciences Tehran Iran

**Keywords:** Chromium, esophageal cancer, mercury, selenium, trace elements, zinc

## Abstract

Studies conducted in China linked selenium deficiency to higher risk of esophageal squamous cell carcinoma (ESCC), but this has not been widely tested outside that selenium‐deficient region. The aim of this study was to investigate the association between selenium and other mineral concentrations in toenails and risk of ESCC in a region with high incidence rates. In this nested case–control study, we identified 222 cases of ESCC from the Golestan Cohort Study, Iran, which has followed up 50,045 participants since enrollment (2004–2008). We randomly selected one control for each case matched by age and sex, using incidence density sampling. We used toenail samples collected at baseline to measure the concentration of selenium, zinc, chromium, mercury, and scandium using instrumental neutron activation analysis. Multivariate adjusted logistic regression models were used to estimate odds ratios (OR) and 95% confidence intervals. Median nail selenium, zinc, chromium, and mercury levels were 1.01, 74.59, 0.77, and 0.018 *μ*g/g in cases and 1.02, 75.71, 0.71, and 0.023 *μ*g/g in controls, respectively. The adjusted odds ratios comparing each fourth quartile of mineral status versus the first quartile were as follows: selenium = 0.78 (95% CI, 0.41–1.49); zinc=0.80 (95% CI, 0.42–1.53); chromium = 0.91 (95% CI, 0.46–1.80); and mercury=0.61 (95% CI, 0.27–1.38), and all trend tests were non–significant. The nail selenium concentration in our controls reflects relatively high selenium status. No evidence of association between selenium or chromium concentrations in toenails and the risk of ESCC was detected in this population.

## Introduction

Esophageal cancer is the sixth most common cause of cancer death in the world. The most divergence in cancer incidence worldwide is seen in esophageal cancer 1, pointing to the possible role of environmental factors in the development of this cancer.

Golestan Province in northeastern Iran is one of the high incidence areas for esophageal cancer, and 90% of the cases are esophageal squamous cell carcinoma (ESCC) 2, which is the most common type of esophageal cancer in the world 3. Ecological studies have reported that selenium concentration is significantly higher and zinc concentration is significantly lower in the soil of high‐risk regions than in low‐risk regions across Golestan province 4, 5, 6, 7. However, studies conducted in central China linked selenium and zinc deficiency to higher risk of ESCC 8, 9.

Selenium is a cofactor of the glutathione peroxidase enzymes, and protects cells against oxidative stress. Moreover, some selenium compounds are antiproliferative and proapoptotic agents and have other anticarcinogenic properties 10. Selenium may prevent cancer by preventing mutation, and decreasing DNA damage via P53 11. Zinc also has antioxidant and proapoptotic properties, and, as a cofactor of metalloenzymes, affects regulation, transcription, and replication of DNA 12, 13. Some compounds of chromium and mercury are suspected to be carcinogens 14, 15, 16. Exposure of animals to chromium in drinking water induced alimentary tract cancer in mice 14.

In a previous study from Golestan Province, we found an inverse association between dietary zinc intake and a U‐shaped association between selenium intake (assessed via food frequency questionnaire) and ESCC risk 17. However, estimating average dietary mineral intake using available food composition data is not always accurate, because the bioavailability of minerals varies with the intake of other dietary components, such as phytate, in the food matrices which can influence their absorption 18 Therefore, the use of biomarkers such as toenails may provide a better estimate of mineral status compared with estimates based on questionnaires in modeling the role of these minerals in ESCC risk 19, 20, 21.

Despite some evidence regarding a possible role for selenium and zinc in ESCC in one high‐risk area of China, this has not been tested outside this one selenium‐deficient region, and the association between chromium or mercury and risk of ESCC has never been investigated, using a reliable biomarker in a prospective study. The aim of this study was, therefore, to investigate the association between prediagnostic toenail concentrations of minerals and the risk of ESCC in a population with high risk of this cancer.

## Methods

### Subjects and study design

The design of the Golestan Cohort Study has been described previously 22. In this cohort study, 50,045 subjects 45 years and older were enrolled between 2004 and 2008 from the eastern three districts of Golestan Province: Gonbad (both urban and rural), Kalaleh (rural), and Aq Qala (rural). The participants provided demographic and dietary data (by a validated food‐frequency‐questionnaire), and provided biospecimens including toenails.

All subjects provided written informed consent before enrollment, and the study was approved by the Institutional Review Boards of the Digestive Disease Research Institute (DDRI) of Tehran University of Medical Sciences and the US National Cancer Institute.

Subjects in the Golestan Cohort Study were followed up for cancer and other major outcomes 22. Participants were asked to contact the cohort team after any new disease diagnosis or hospitalization. Also, subjects were interviewed by phone annually and a short health questionnaire was administered. Each report of cancer diagnosis was followed by a home or medical center visit. The cohort team collected all clinical reports, hospital records, tumor samples, and pathology reports which were available. Any report of pathology was confirmed by an Endpoint Review Committee. The cohort subject list was also checked against the Golestan Population‐based Cancer Registry (GPCR), which covers all Golestan Province and is one of the high‐quality registries included in Cancer Incidence in Five Continents 22. Loss to follow‐up in the cohort is negligible (<1%).

For each case, a control was selected randomly via incidence density sampling without replacement 23. Eligible controls were free of esophageal or other digestive tract cancers at the time of case diagnosis, and were risk‐set matched to cases individually on age (within 2 calendar years) and sex.

### Mineral concentration measurements

During the baseline enrollment examination, subjects were asked to provide a toenail sample. Nail specimens were placed in labeled plastic bags and stored at room temperature. Toenails were provided by 99.9% of participants.

The concentrations of selenium, zinc, chromium, and mercury were measured by instrumental neutron activation analysis (INAA) at the University of the Missouri Research Reactor. Details of the method, which has been used for selenium and other minerals, has been published before 21.

For quality control, the samples were coanalyzed with NIST SRM 1577 Bovine Liver, NIST SRM 1571 Orchard Leaves, and NCS DC 73347 Hair. The measured values of selenium, chromium, zinc, and mercury in these samples agreed well with the accepted values.

Concentrations of scandium and aluminum in toenails were also measured because they are understood to reflect terrestrial material, soil, or dust that is adherent to the nail. Selenium, zinc, and mercury were not correlated with these terrestrial indicators, but chromium was correlated with the concentrations of scandium and aluminum. Therefore, we used the residual method and regressed the concentration of chromium on the concentration of scandium, added back the mean and used this residually adjusted value. Given the need to perform this procedure to decrease the potential effect of terrestrial contamination, the results of the chromium analysis should be interpreted with caution.

Two hundred and twenty‐nine toenail samples had sufficient mass to allow preparation of a duplicate sample for analysis. The mean difference between duplicate samples did not differ significantly from zero. The coefficients of variation for duplicate samples were 2.3% for selenium, 4.1% for zinc, 17.1% for chromium, and 27% for mercury.

### Statistical analysis

Baseline demographic data for ESCC cases and controls were compared using chi‐square tests for categorical variables and Kruskal–Wallis tests for continuous variables. Partial correlations were used to assess the predictors of nail element concentrations.

Associations between toenail minerals and risk of ESCC were examined using multivariate‐adjusted logistic regression. Since we matched individually on age and sex, we initially used conditional regression models. However, we observed associations between selenium and mercury concentrations and district of residence. Since the participants of our study were from Gonbad (urban, *n* = 97, and rural, *n* = 169), Kalaleh (only rural, *n* = 166), and Aq Qala (only rural, *n* = 11), we made a composite variable for place of residence using district and rural/urban status and created four categories: Gonbad rural, Gonbad urban, Kalaleh, and Aq Qala. For the logistic regression model results, which are presented, we broke the matching to allow better control for confounding by place of residence and used unconditional logistic regression for analysis. All models included the matching factors (age and sex) and the composite place of residence variable. The results of the conditional logistic regression models did not differ meaningfully from those of the unconditional logistic regression models, but the unconditional models provided better precision.

Mineral concentrations were divided into quartiles using nail concentrations among controls, and median values of each quartile were used to test for trend. For the logistic regression models, we scaled the continuous metrics of the mineral concentrations to one half of the interquartile range among controls ((75^th^ percentile‐25^th^ percentile)/2), which is easier to compare to the step‐wise differences between quartiles. The continuous ORs should be interpreted as increase of 0.11 *μ*g/g for selenium, 11.15 *μ*g/g for zinc, 0.40 *μ*g/g for chromium, and 0.01 *μ*g/g for mercury.

Suspected confounders were tested in the unconditional logistic regression models including age, sex, place of residence (Gonbad rural, Gonbad urban, Kalaleh, and Aq Qala), smoking (pack‐years), opium use (ever, never), socioeconomic status (determined by a composite score) 24, formal education (none, any), ethnicity (non‐Turkmen, Turkmen), body mass index (BMI) (<18.5, 18.5 to <25, 25 to <30, ≥30), family history of esophageal or gastric cancer in first‐degree relatives (yes, no), physical activity level at work (irregular nonintense, regular nonintense, irregular intense, or regular intense) 17, and the daily intake of fruits and vegetables (grams/day). Since alcohol is not a risk factor in this region due to the very low prevalence of alcohol use (4 percent) 25, we did not adjust for drinking alcohol.

Interactions were investigated by multiplying each mineral concentration by each potential effect modifier and the resulting models were tested using the likelihood ratio test. In sensitivity analyses, we excluded subjects with BMI less than 18.5 or more than 35 kg/m^2^. We stratified our analysis by the follow‐up time (time from enrollment to diagnosis: ≤2 years, >2–5 years, and > 5 years). Potential nonlinear associations between each mineral concentration and risk of ESCC were evaluated using restricted spline models**.** The data were analyzed using STATA software (version 13, STATA Corp, College Station, TX). All tests of statistical significance were two‐sided and p‐values <0.05 were considered statistically significant.

## Results

### Subjects characteristics

At the time of selection, there were 222 eligible ESCC cases and 222 controls in the study, but a single case did not provide a toenail sample. Baseline characteristics for the remaining 221 ESCC cases and 222 controls are shown in Table 1.

**Table 1:** Baseline characteristics of esophageal squamous cell carcinoma case and control participants in the golestan cohort study

	ESCC Case subjects (*N *=* *221)	Control subjects (*N *=* *222)	*P* value
Age, years a	60.2 ± 9.3	59.1 ± 9.3	0.23
Male b	120 (54.3)	120 (54.1)	0.96
BMI^a^, kg/m^2^	23.1 ± 4.4	26.8 ± 6.1	<0.001
Smoking, pack‐year^a^	5.7 ± 19.6	2.9 ± 9.1	0.30
SES^b^			<0.001
Low	106 (48.0)	60 (27.0)	
Low‐ Medium	54 (24.4)	55 (24.8)	
Medium‐ High	41 (18.5)	58 (26.1)	
High	20 (9.0)	49 (22.1)	
Family history, positive^b^	55 (24.9)	43 (19.3)	0.16
Place of residence b			<0.001
Gonbad urban	17 (7.7)	81 (36.5)	
Gonbad rural	97 (43.9)	71 (32.0)	
Kalaleh	98 (44.3)	68 (30.6)	
Aq Qala	9 (4.1)	2 (0.9)	
Ethnicity, Turkmen^b^	182 (82.3)	167 (75.2)	0.07
Opium use^b^	57 (25.8)	37 (16.7)	0.02
No formal education^b^	186 (84.1)	161 (72.52)	0.003
Physical activity			0.03
Irregular, nonintense	154 (69.7)	126 (56.8)	
Regular, nonintense	48 (21.7)	65 (29.3)	
Irregular or regular, intense	19 (8.6)	30 (13.5)	
Fruit intake, grams/day^a^	143.3 ± 168.4	168.7 ± 123.0	<0.001
Vegetable intake, grams/day^a^	176.5 ± 87.3	201.4 ± 101.9	0.02
Baseline nail mineral levels (*μ*g/g), median (interquartile range)			
Selenium	1.009 (0.89–1.13)	1.020 (0.92–1.14)	0.18
Zinc	74.59 (62.60–83.67)	75.71 (63.71–86.89)	0.22
Chromium	0.77 (0.47–1.44)	0.71 (0.42–1.13)	0.007
Mercury	0.018 (0.01–0.03)	0.023 (0.01–0.05)	0.002

a Mean ± SD.

b Percent.

### Mineral concentrations

The median and interquartile range of nail concentration of each mineral are shown in Table 1. The concentration of selenium and zinc did not differ significantly between cases and controls. Incident ESCC cases had lower baseline nail concentrations of mercury and higher concentrations of chromium (Table 1).

Partial correlations were used to investigate the association between the concentration of each mineral and each suspected risk factor or other minerals. The percent of squared partial correlations of minerals have been shown in Table S1.

The concentration of selenium in nails was different in each place of residence (*p* = 0.008). The median (interquartile range) nail concentration in Gonbad rural was 1.056 (0.901–1.174) *μ*g/g, in Gonbad urban was 1.019 (0.947–1.128), in Kalaleh was 1.004 (0.897–1.130) *μ*g/g, and in Aq Qala was 0.920 (0.797–1.044) *μ*g/g.

### Mineral concentrations and cancer risk

In the fully adjusted quartile analyses, there were no statistically significant differences in any of the mineral concentrations by case status (Table 2). In the fully adjusted continuous analyses, the concentrations of selenium, zinc, and chromium were not associated with case status, but the mercury concentration was inversely associated with risk of ESCC (HR = 0.88, 95% CI = 0.79–0.99). Further adjustment by usual consumption of fish had little effect on the point estimate for mercury, but did make the association nonsignificant (HR = 0.89, 95% CI = 0.79–1.00, *P* = 0.052). Adjustment for nail mass and year or season of nail collection did not change the point estimates.

**Table 2:** Associations of nail mineral concentrations and esophageal squamous cell carcinoma in the Golestan Cohort Study

	Quartile analysis				Continuous analysis^a^		
	Q1	Q2	Q3	Q4	*P* for trend		*P*
Selenium							
Cases	70	45	54	52			
OR^b^ (95% CI)	1	0.65 (0.39–1.11)	0.77 (0.46–1.29)	0.76 (0.45–1.27)	0.38	0.94 (0.84–1.06)	0.35
OR^c^ (95% CI)	1	0.90 (0.48–1.69)	0.92 (0.49 –1.74)	0.78 (0.41–1.49)	0.48	0.94 (0.80–1.09)	0.40
Zinc							
Cases	64	55	55	47			
OR^b^ (95% CI)	1	0.87 (0.52–1.47)	0.86 (0.51–1.44)	0.75 (0.44–1.27)	0.29	0.92 (0.82–1.03)	0.13
OR^c^ (95% CI)	1	0.75 (041–1.37)	0.90 (0.49–1.67)	0.80 (0.42–1.53)	0.62	0.96 (0.83–1.10)	0.54
Chromium							
Cases	45	55	50	71			
OR^b^(95% CI)	1	1.24 (0.72–2.14)	1.11 (0.64–1.92)	1.61 (0.95–2.72)	0.09	1.1 (1.01–1.20)	0.04
OR^c^ (95% CI)	1	0.88 (0.44–1.76)	0.63 (0.31–1.25)	0.91 (0.46–1.80)	0.97	1.03 (0.93–1.13)	0.60
Mercury							
Cases	69	63	66	23			
OR^b^ (95% CI)	1	0.93 (0.56–1.54)	0.97 (0.59–1.61)	0.33 (0.18–0.61)	<0.001	0.84 (0.77–0.92)	0.000
OR^c^ (95% CI)	1	0.95 (0.53–1.70)	1.59 (0.87–2.91)	0.61 (0.27–1.38)	0.29	0.88 (0.79–0.99)	0.03

a should be interpreted as increase of 0.11 *μ*g/g for selenium, 11.15 *μ*g/g for zinc, 0.40 *μ*g/g for chromium, and 0.01 *μ*g/g for mercury.

b Crude model.

c Adjusted for age (years), sex (M, F), place of residence (Gonbad urban, Gonbad rural, Kalaleh, Aq Qala), smoking (pack‐years), socioeconomic status (low, low‐medium, medium‐high, high), ethnicity (non‐Turkmen, Turkmen), opiate use (never, ever), body mass index (<18.5, 18.5 to <25, 25 to <30, >=30), education (no formal, formal education), physical activity (irregular nonintense, regular nonintense, irregular or regular intense), family history (positive, negative), fruit intake (g/d), and vegetable intake (g/d); ORs (95% CI) were calculated using an unconditional logistic regression models.

There was no interaction between mineral content and gender, age, education, place of residence, or ethnicity and risk of ESCC. We excluded subjects with BMI less than 18.5 or more than 35 kg/m^2^, because the mean BMI was significantly lower in case subjects, but the results did not change significantly. Stratifying by follow‐up time, the adjusted hazard ratios for zinc was 0.26 (95% CI = 0.07–0.97), and for chromium was 0.22 (95% CI = 0.05–0.96), for ESCC cases diagnosed more than 5 years after enrollment (Table S2).

Lastly, we explored the possibility of a nonlinear relationship between each mineral and risk of ESCC using restricted cubic splines, however we found no evidence of such a relationship (*P*‐values for a nonlinear association were: selenium = 0.58, zinc = 0.90, chromium = 0.50, and mercury = 0.13).

## Discussion

In our case–control study nested within the Golestan Cohort, selenium and chromium nail concentrations were not significantly associated with risk of ESCC. There was no effect modification of BMI, ethnicity, education, opium, or smoking in the results.

Mark et al.*,* showed an inverse association between serum selenium levels and risk of ESCC in the Nutrition Intervention Trial (NIT) cohort in a selenium‐deficient area in China 8. In another previous study, Steevens et al., showed an inverse association between toenail selenium concentrations and the risk of ESCC in the Netherland Cohort Study 26. However, the median toenail selenium was 0.55 *μ*g/g in the Dutch population, compared with 1.01 *μ*g/g in Golestan. Thus, the lack of association in our study may be related to the fact that very few individuals in our study were selenium‐deficient. In a previous study of dietary selenium, we found a statistically significant nonlinear U‐shaped association between selenium intake and the risk of ESCC in the Golestan Cohort population 17. However, selenium intake did not correlate with toenail measurements in our study (*r* = 0.02). Similar discrepancies have been reported in other studies. In the ORDET study, toenail and dietary selenium were not correlated, (*r* = 0.02; *P* = 0.72) 27, and in Ovaskainen et al.*,* they were weakly correlated (*r* = 0.18, *P* ≤ 0.05) 28. Longnecker et al.*,* reported the concentration of selenium in a single specimen of whole blood, serum, or toenails served reasonably well as a measure for ranking subjects according to long‐term selenium intake but provided only a rough estimate of intake for each subject 29. We acknowledge the potential for residual confounding from other (unmeasured) dietary components which are correlated with selenium intake. There might be differences in metabolism in different subjects.

An ecologic study in Golestan Province by Keshavarzi et al. reported that the selenium concentrations in soil, grain, and drinking water increase from the low‐risk area to the high‐risk area for ESCC 4. The relative concentrations of selenium in nails that we measured (nail concentration in Gonbad = 1.034 *μ*g/g, and in Kalaleh=1.004 *μ*g/g) were compatible with the relative concentrations of selenium in soil in the Keshavarzi et al. study (soil concentration in Gonbad = 1.22 *μ*g/g, and in Kalaleh = 0.77 *μ*g/g). In another ecologic study, the median serum selenium level in subjects who live in Golestan Province was higher than that in other low‐risk areas for esophageal cancer in Iran 7. The selenium intake among cohort participants in Golestan was greater than the US RDA (55 *μ*g/day) in our previous study 17. Nail selenium concentrations among controls in our study were high in comparison with previous studies which targeted other diseases in other population (Table 3) 26, 27, 28, 29, 30, 31. Our findings suggest that selenium deficiency is not a risk factor for the high incidence of ESCC in northeastern part of Iran.

**Table 3:** Selenium concentrations of toenail samples among controls in previous studies

The place of the Study	The name of the Study	Median nail selenium concentrations (*μ*g/g)	Mean nail selenium concentrations (*μ*g/g)
Iran (current study)	GCS^a^	1.02 (0.92–1.14)	
US, MD [30]	CLUE II^b^	0.79 (0.70–0.87)	
US [30]	HPFS^c^	0.80 (0.73–0.94)	
Netherlands [26]	NLCS^d^	0.55 (0.48–0.60)	
US [30]	SELECT^e^	0.88 (0.77–0.99)	
Italy [27]	ORDET^f^		0.96
China [31]			0.47 (range=008–4.22)
Finland [28]			0.47 ± 0.09

a GCS = Golestan Cohort Study.

b CLUE II = Campaign against Cancer and Stroke.

c HPFS = Health Professionals Follow‐up Study.

d NLCS = Netherlands Cohort Study.

e SELECT = Selenium and Vitamin E Cancer Prevention Trial.

f Hormones and Diet in the Etiology of Breast Cancer.

Our previous analysis of dietary intake of zinc in this population showed an inverse association between zinc intake and risk of ESCC 17. The difference in the results of this previous dietary intake analysis and the current analysis of nail concentrations could be due to different metabolism of zinc in different subjects, or to the confounding effect of other (unmeasured) agents which are correlated with intake of zinc. Keshavarzi et al. found that total zinc concentrations in soil and grain samples decreased from low‐ to high‐risk areas for ESCC in Golestan 4, and Abnet et al., found an inverse association between tissue zinc concentrations in normal esophageal tissue and risk of ESCC in a prospective study in China 9. We also found an inverse association between nail zinc concentration and ESCC in subjects who were followed more than 5 years. This results could be due to sparse data or may reflect that zinc concentrations could have been due to reverse causation. We are not aware of any previous prospective study which has investigated the association between toenail zinc status and ESCC 32. Nouri et al., showed that the nail zinc concentration was lower in 20 cases than 80 controls in a case–control study 33, and in the NIT intervention trial in China, the group that received oral zinc and retinol supplements experienced no reduction in ESCC risk 34.

This study is the first to evaluate the effect of mercury and chromium on risk of ESCC. Some compounds of these two minerals are suspected to be carcinogens 14, 15, 16. Chromium may cause mutations and chromosomal breaks and exposure of animals to chromium in drinking water can induce alimentary tract cancer in mice 14. Our results showed no association between nail chromium concentrations and ESCC risk. Few other studies have measured chromium in toenails. The concentration of chromium in our study (0.71 *μ*g/g) was more than that reported in the FINBAR study conducted in Ireland (0.55 *μ*g/g) 35. Surprisingly, we also found an inverse association between toenail chromium concentration and risk of ESCC. The number of cases in this analysis was small. We are not aware of any study to support a protective association with chromium and we therefore conclude that this a chance finding.

Methylmercury compounds have been reported to be associated with lung, brain, and prostate cancer 15. In this study, although the highest quartile of toenail mercury was not associated with risk of ESCC, increasing toenail mercury concentration as a continuous variable was inversely associated with ESCC. This inverse association may reflect the protective effect of fish consumption (the primary dietary source of mercury). After adjusting the model for fish consumption, the association remained protective, but was no longer statistically significant. We caution that the observed association is based on small case numbers. Also of note, the mean concentration of mercury among controls in this study was very low (0.04 ± 0.05 *μ*g/g) compared to those reported in the Nurse's Health Study (0.33 ± 0.63 *μ*g/g) and the Health Professionals Follow‐up Study (excluding dentists) (0.44 ± 0.47 *μ*g/g) in the US 36 and the FINBAR case‐control study (0.11 ± 0.23 *μ*g/g) in Ireland 35.

One of the strengths of this study is that toenails were collected at the beginning of the cohort study, so the mineral concentrations are prediagnostic, and 99% of the cohort population provided nail samples. Toenail measurements are believed to represent a marker of exposure during last year prior to clipping. A limitation of our study is that soil levels of selenium differ across the region and having a larger number of cases across the different regions would allow us more power to look at the association in high and low selenium regions. We collected nail samples at baseline which reflect the mineral status at baseline of the study, we were not able to investigate the changes in diet during the follow‐up. We suggest that future studies evaluate the changes in element concentrations in nail samples over time.

In summary, this study in a high‐risk area of ESCC, showed no evidence of association between toenail selenium, or chromium and risk of ESCC.

## Conflict of Interest

The authors have declared no conflicts of interest.

## Supporting information


**Table S1.** Correlations between nail mineral concentrations and suspected risk factors for esophageal squamous cell carcinoma in the controls from the Golestan Cohort Study.**Table S2.** Associations of nail mineral concentrations and esophageal squamous cell carcinoma stratified by follow‐up time.
