# Supplementary material for: Toenail mineral concentration and risk of esophageal squamous cell carcinoma, results from the Golestan Cohort Study
Source: Cancer Med. 2017 Nov 10;6(12):3052–9. doi: 10.1002/cam4.1247 (PMC5727321; doi:10.1002/cam4.1247)
Supplement: Supplementary file 1 — Table S1. Correlations between nail mineral concentrations and suspected risk factors for esophageal squamous cell carcinoma in the controls from the Golestan Cohort Study.Table S2. Associations of nail mineral concentrations and esophageal squamous cell carcinoma stratified by follow‐up time. [file CAM4-6-3052-s001.docx]

| Supplementary Table 1. Correlations between nail mineral concentrations and suspected risk factors for esophageal squamous cell carcinoma in the controls from the Golestan Cohort Study | | | | |
| --- | --- | --- | --- | --- |
|  | **Partial correlation squared (percent)** | | | |
| Variable | **Selenium** | **Zinc** | **Chromium** | **Mercury** |
| Sex | 6.67** | 1.28 | 2.48* | 2.37* |
| Age | 0.00 | 1.82 | 0.02 | 0.01 |
| education | 0.15 | 0.30 | 3.76* | 2.53* |
| BMI, kg/m^2^ | 0.81 | 0.59 | 1.90 | 0.04 |
| Place of residence | 2.21* | 0.12 | 1.06 | 2.62* |
| Opium | 5.30** | 0.24 | 0.01 | 0.19 |
| smoking | 0.09 | 0.25 | 1.47 | 0.55 |
| Physical activity | 0.04 | 0.33 | 2.43* | 0.14 |
| Vegetable intake | 0.01 | 0.26 | 1.68 | 0.03 |
| Fruit intake | 0.24 | 0.14 | 0.25 | 1.02 |
| Ethnicity | 0.35 | 0.45 | 0.56 | 0.68 |
| Socioeconomic status | 1.31 | 1.12 | 2.1* | 2.71* |
| Other minerals |  |  |  |  |
| Selenium | - | 0.78 | 0.36 | 0.12 |
| Zinc | 0.78 | - | 2.28* | 5.89** |
| Chromium | 0.36 | 2.28* | - | 3.78* |
| Mercury | 0.12 | 5.89** | 3.78* | - |

*p<0.05

**p<0.001

| Supplementary Table 2. Associations of nail mineral concentrations and esophageal squamous cell carcinoma stratified by follow-up time | | | | | | |
| --- | --- | --- | --- | --- | --- | --- |
|  |  | **Quartile analysis^1^** | | | | **Continuous analysis ^2^** |
|  | **Cases** | **Q1** | **Q2** | **Q3** | **Q4** |  |
| Selenium |  |  |  |  |  |  |
| <2 years follow-up | 52 | 1 | 0.80 (0.21-3.00) | 0.94 (0.24-3.67) | 1.19 (0.26-5.41) | 0.88 (0.60-1.29) |
| 2-5 years follow-up | 80 | 1 | 0.62 (0.18-2.10) | 0.87 (0.28-2.73) | 0.75 (0.24-2.35) | 0.92 (0.96-1.23) |
| >5 years follow-up | 89 | 1 | 3.16 (0.80-12.5) | 1.89 (0.49-7.39) | 1.25 (0.34-4.58) | 1.11 (0.84-1.46) |
| Zinc |  |  |  |  |  |  |
| <2 years follow-up | 52 | 1 | 1.15 (0.30-4.48) | 1.41 (0.32-6.19) | 1.93 (0.46-8.09) | 1.10 (0.81-1.47) |
| 2-5 years follow-up | 80 | 1 | 1.15 (0.40-3.35) | 1.30 (0.42-3.95) | 1.02 (0.27-3.80) | 1.03 (0.77-1.38) |
| >5 years follow-up | 89 | 1 | 0.39 (0.11-1.35) | 0.65 (0.19-2.19) | 0.26 (0.07-0.97) | 0.75 (0.55-1.03) |
| Chromium |  |  |  |  |  |  |
| <2 years follow-up | 52 | 1 | 0.74 (0.16-3.44) | 0.39 (0.09-1.74) | 1.34 (0.29-6.13) | 1.15 (0.91-1.46) |
| 2-5 years follow-up | 80 | 1 | 0.85 (0.22-3.24) | 1.27 (0.34-4.76) | 1.27 (0.35-4.61) | 1.04 (0.87-1.24) |
| >5 years follow-up | 89 | 1 | 0.58 (0.15-2.56) | 0.26 (0.06-1.18) | 0.22 (0.05-0.96) | 0.96 (0.85-1.09) |
| Mercury |  |  |  |  |  |  |
| <2 years follow-up | 52 | 1 | 1.61 (0.46-5.67) | 2.02 (0.58-7.04) | 0.64 (0.09-4.40) | 0.87 (0.70-1.08) |
| 2-5 years follow-up | 80 | 1 | 1.74 (0.55-5.51) | 5.41 (1.49-19.6) | 0.37 (0.05-2.73) | 0.90 (0.68-1.19) |
| >5 years follow-up | 89 | 1 | 0.41 (0.12-1.45) | 0.34 (0.09-1.24) | 0.33 (0.08-1.40) | 0.81 (0.65-1.02) |
| ^1^ Adjusted for age (years), sex (M, F), place of residence (Gonbad urban, Gonbad rural, Kalaleh, Aq Qala), smoking (pack-years), socioeconomic status (low, low-medium, medium-high, high), ethnicity (non-Turkmen, Turkmen), opiate use (never, ever), body mass index (<18.5, 18.5 to <25, 25 to <30, >=30), education (no formal, formal education), physical activity (irregular non-intense, regular non-intense, irregular or regular intense), family history (positive, negative), fruit intake (g/d), and vegetable intake (g/d); ORs (95% CI) were calculated by using an unconditional logistic regression models  ^2^ should be interpreted as increase of 0.11 µg/g for selenium, 11.15 µg/g for zinc, 0.40 µg/g for chromium and 0.01 µg/g for mercury | | | | | | |
